# Windblown sand hazards risk assessment along the highways based on GIS-game theory combination weight

**DOI:** 10.1371/journal.pone.0292263

**Published:** 2024-02-08

**Authors:** Liangying Li, Lele Lv, Zhizhong Tao, Wenhua Yin, Qi Li, Zhenqiang Wang

**Affiliations:** 1 School of Civil Engineering, Lanzhou Jiaotong University, Lanzhou, China; 2 Ningxia Highway Survey and Design Institute Co Ltd, Yinchuan, China; University 20 Aout 1955 skikda, Algeria, ALGERIA

## Abstract

Windblown sand hazards seriously threaten the safe operation of highways in desert areas. Reasonable risk assessment can provide the basis for windblown sand hazards prevention and risk reduction. To facilitate the formulation of better windblown sand hazards prevention and reduction strategies, a new windblown sand hazards risk assessment model along the highways was proposed, in which seven evaluation indicators were selected from danger of the hazard-causing factors, vulnerability of the hazard-forming environment, and the vulnerability of the hazard-bearing body. The model was established based on the combination weighting method of game theory, and the risk map was generated based on the GIS platform. Finally, the model was applied to the windblown sand hazards risk assessment along the Wuhai-Maqin Highway. The result showed that the risk of the windblown sand hazards along the Wuhai-Maqin Highway is mainly medium, low, and very low. High and very high risk windblown sand hazards sections account for only 33% of the total length of the highway. The high and very high risk highway sections of the windblown sand hazards are mainly distributed in the hinterland of shifting dunes area and near the horizontal curve with a small radius in the flat sandy land area. By comparing with the real information of windblown sand hazards along the highway, correlation was up to 85.93%, which verified the accuracy of the model. The model can be applied to windblown sand hazards risk assessment along the highways.

## 1 Introduction

There are more than 7,900 kilometers of desert highways in China, which are widely distributed in Xinjiang Province, Inner Mongolia Province, Ningxia Province, and Gansu Province. Furthermore, more than 2,300 kilometers of desert highways are exposed and harmed by wind-sand. With the continuous advancement of “the Belt and Road” initiative and the improvement of the highway transportation network, the mileage of highways in the desert area has increased year by year, and the windblown sand hazards along the highway has become increasingly intensified. It seriously affects the design, construction, and operation of the highway [1, 2]. Accurate assessment of windblown sand hazards risk along highways is of great significance for windblown sand hazards prevention and smooth highway.

In recent years, scholars have continued to innovate in the study of assessment of windblown sand hazards risk. Wang et al. [3] and Xu et al. [4] used mathematical-statistical methods to evaluate the risk of windblown sand hazards based on historical disaster statistical data. However, this method is not widely applicable due to the reliance on a large amount of statistical data. After the “three-factor theory” of disaster formation was proposed [5], the multi-criteria decision analysis (MCDA) based on it has gradually become the primary method of natural hazard risk assessment. And with the development of computer technology, many scholars have made use of GIS technology to visualise the results of the assessment. Yue et al. [6, 7] obtained the distribution of high-risk lakes and cities of windblown sand hazards through GIS technology after calculating the indicator weights with AHP. Guan et al. [8] and Yang et al. [9] obtained the distribution map of windblown sand hazards risk after calculating the subjective weight and objective weight by AHP and the entropy weight method (EWM), respectively. Rezaei et al. [10] and Gholami et al. [11] mapped the distribution of potential factors controlling soil wind erosion hazards through GIS technology and used neural network modelling to achieve the risk assessment of soil wind erosion hazards in Fars province and Semnan province of Iran.

For the windblown sand hazards along the highway, scholars also make risk assessments based on MCDA. Wang et al. [12] made MCDA through the danger of hazard-causing factors and established a fuzzy set to grade the results after directly assigning weights to indicators based on expert experience. Li et al. [13] constructed an assessment model of windblown sand hazards along the highway based on the “three-factor theory”. After the indicators weights were determined by the AHP, the highway section with different windblown sand hazards risk grades were obtained. Although the research on windblown sand hazards risk assessment along the highway has been enriched by the above studies, there are still the following deficiencies:

1. The visualisation of the assessment results is relatively poor as the windblown sand hazards risk assessment along the highways is rarely combined with GIS effectively.
2. For MCDA, the determination of indicator weights is crucial. However, in previous studies, the determination of weights lacked rationality due to the fact that the weights of the indicators were determined directly by expert experience or calculated through a single method.

Game theory can effectively increase the rationality of indicator weights. The combination of game theory and GIS can make the evaluation results more reasonable and intuitive, and has been well applied in the risk assessment of water erosion hazards [14] and dust storm hazards [15–17].

Therefore, in this paper, to make the assessment results more intuitive and reasonable, taking the wind-sand section of the Wuhai-Maqin Highway as an example, the indicators system and the assessment model of windblown sand hazards risk along the highway according to the “three-factor theory” and the combined weighting of the game theory was established. The scope and risk level of windblown sand hazards are visualized based on the GIS platform. The results of the study can more accurately and quickly realise the windblown sand hazards risk assessment along the desert highways, which can provide a reference for decision makers to formulate windblown sand hazards prevention and reduction strategies.

## 2 Study area

### 2.1 Overview

**Fig 1:**
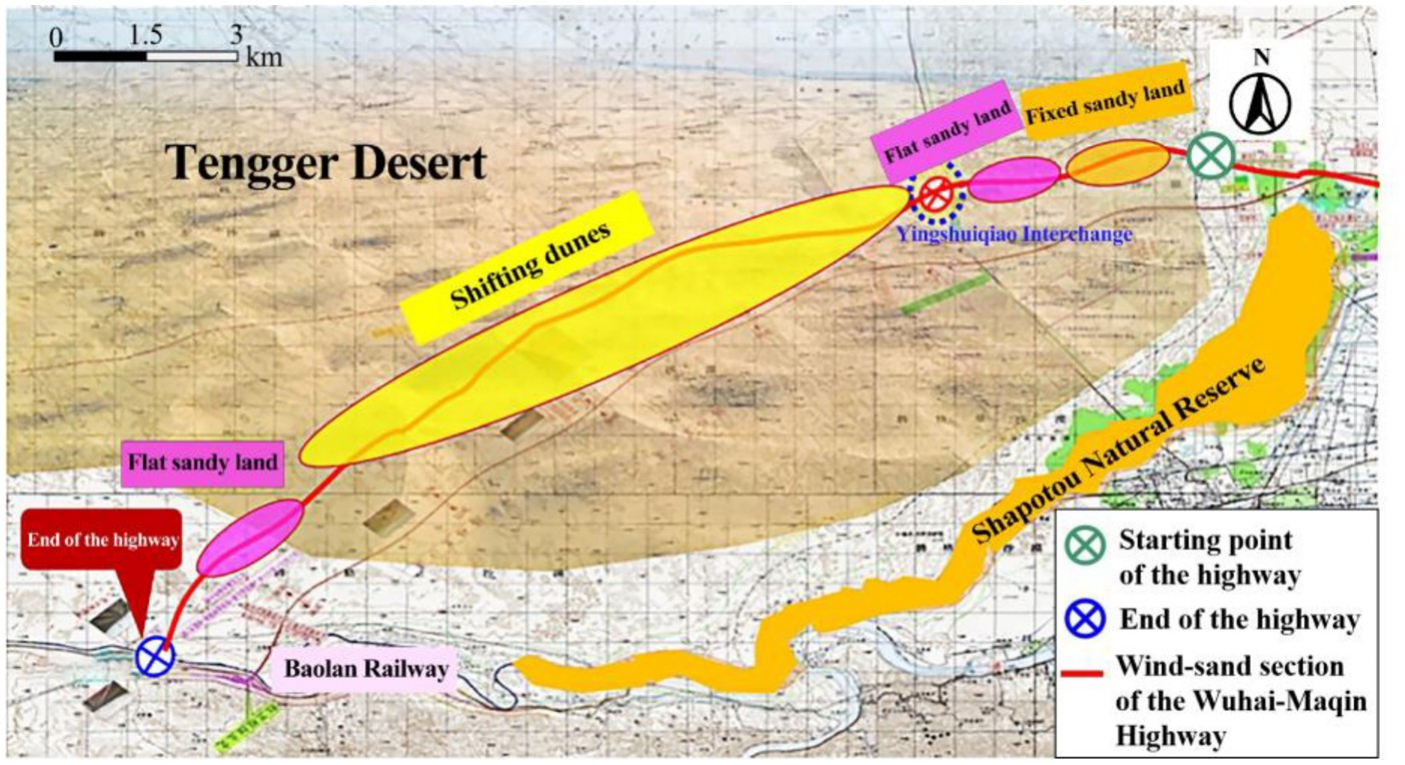
Geographical environment along the wind-sand section of the Wuhai–Maqin Highway.

The wind-sand section of the Wuhai-Maqin Highway is located in the Tengger Desert, where shifting dunes, flat sandy land, and fixed sandy land are widely distributed (Fig 1). The climate of the area is the temperate continental extreme arid climate, with sparse rainfall, strong evaporation, and intense wind, which can easily lead to windblown sand hazards [18–20].

### 2.2 Data

**Fig 2:**
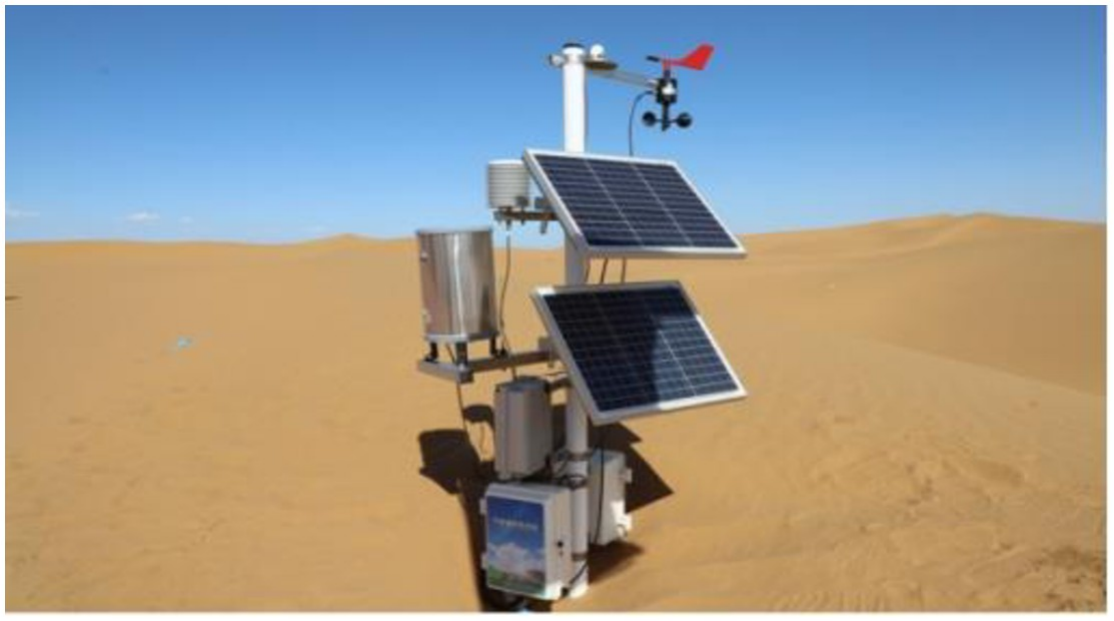
Automatic weather station.

The meteorological data come from 7 field automatic weather stations (Fig 2) set up along the wind-sand section of Wuhai–Maqin Highway from east to west. Its observation height is 2m, and the collection frequency is 5min/time. It is used to monitor the wind direction, wind speed, precipitation, temperature, and other meteorological data. A meteorological digital management platform was established to collect statistics on the data. Before the construction of the highway, the design unit carried out a detailed survey of the type of sandy land and the vegetation along the highway and prepared a corresponding survey report, from which we can get the distribution of the type of sand land and the vegetation cover along the highway. The highway route data were also obtained from the design unit.

## 3 Methodology

**Fig 3:**
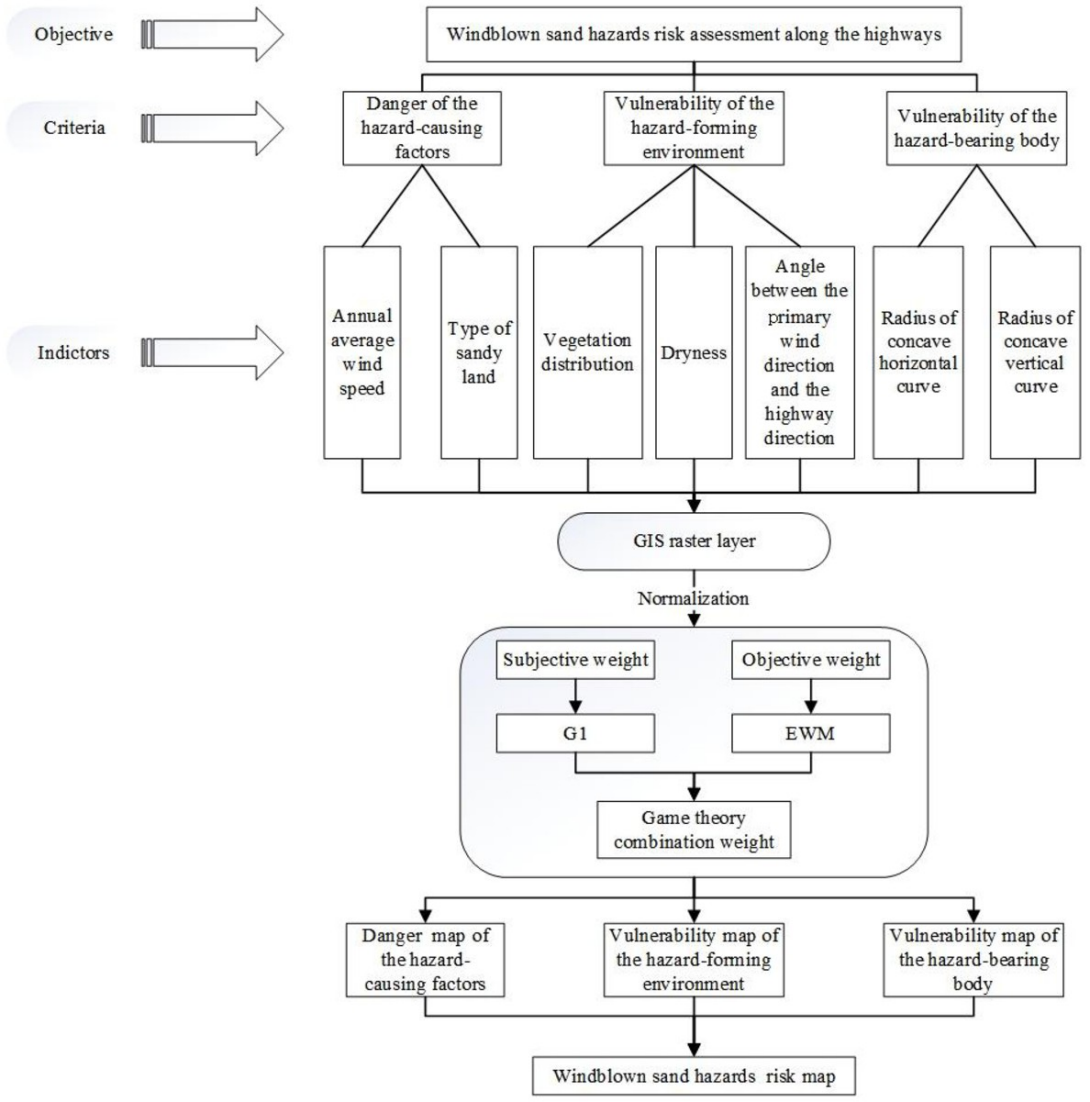
Framework for windblown sand hazards risk assessment along the highways.

The framework for windblown sand hazards risk assessment along the highways is shown in Fig 3. Windblown sand hazards risk is considered the product of the danger of the hazard-causing factors, the vulnerability of the hazard-forming environment, and the vulnerability of the hazard-bearing body [21]. The risk of windblown sand hazards was taken as the evaluation objective. The danger of the hazard-causing factors, the vulnerability of the hazard-forming environment, and the vulnerability of the hazard-bearing body were taken as the evaluation criteria, and the evaluation indicators were determined according to the evaluation criterion. The importance of each indicator to the evaluation results was reflected by weight. The subjective weight was determined by the G1 method, and the objective weight was determined by the entropy weight method (EWM). The combined weight of each index was calculated by the game theory method.

In Arcmap 10.2, the buffer zone of 2km is made on each side centered on the highway as a range for processing data. The evaluation indicators were processed using ArcMap10.2 and each indicator layer was made into a raster layer. Each raster layer has a resolution of 58.9m. The indicators were classified with reference to existing research results. Then, the indicators were then normalized to remove the effect of order of magnitude differences among them. After weighted superposition, the danger map of the hazard-causing factors, the vulnerability map of the hazard-forming environment, and the vulnerability map of the hazard-bearing body were generated. Ultimately, after multiplying the results of the three maps, the windblown sand hazards risk map was generated, which was divided into five levels: very low, low, medium, high, and very high.

### 3.1 Evaluation indicators

Referring to the previous research on windblown sand hazards analysis, based on the three-factor theory and the characteristics of the highways, seven evaluation indicators were selected and classified, as shown in Table 1.

**Table 1:** Evaluation indicators and classification.

		Low	Medium	High
Danger of the hazard-causing factors	Annual average wind speed(m/s)	<6	6~6.5	>6.5
	Type of sandy land	Fixed sandy land	Flat sandy land	Shifting dunes
Vulnerability of the hazard-bearing environment	Vegetation distribution	Exuberant	Medium	Sparse
	Dryness	>7	5~7	<5
	Angle between the primary wind direction and the highway direction	0~30°	30~60°	60~90°
Vulnerability of the hazard-bearing body	Radius of concave horizontal curve(m)	>5500	1000~5500	<1000
	Radius of concave vertical curve(m)	>5500	6000~10000	<6000

#### 3.1.1 Evaluation indicators for danger of the hazard-causing factors

Windblown sand hazards are essentially secondary wind hazards, the extent of which is closely related to the intensity of wind-sand flows. As a typical gas-solid two-phase flow, the sand source is the material basis for its formation, and the strong wind is the dynamic condition for its formation [22]. The annual average wind speed are not only easy to count, but also reflect the overall strength of wind in the region over the course of a year. There are differences in the amount of sand sources that different types of sandy land can provide for wind-sand activities. Therefore, the annual average wind speed and the type of sandy land were selected as the evaluation indicators for the danger of the hazard-causing factors.

According to the data of the weather stations along the highway, the annual average wind speed was calculated, and then the annual average wind speed distribution of the study area was obtained by the inverse distance weight(IDW). Based on the results of the site survey, the type of sandy land was divided into shifting dunes, flat sandy land and fixed sandy land.

#### 3.1.2 Evaluation indicators for vulnerability of the hazard-forming environment

The intensity of wind-sand flows is also influenced by environmental factors. In general, the higher the vegetation cover, the correspondingly greater the surface roughness. The influence of friction makes the wind speed near the surface smaller, and the less likely it is to form wind-sand flows [23, 24]. The wetter the climate, the greater the moisture content in the surface soil. On the one hand, it increases the cohesion of the particles, thereby enhancing the resistance of the surface soil to wind erosion, on the other hand, the moisture in the soil may increase the survival rate of surface vegetation [25]. The dryness integrates the precipitation and temperature data of the region, which can better reflect the humid conditions of the regional climate [26, 27]. In addition, the intensity of wind-sand flows along the highway is closely related to the angle between the wind direction and the highway [28]. Therefore, vegetation distribution, dryness, and Angle between the primary wind direction and the highway direction were selected as the evaluation indicators for the vulnerability of the hazard-forming environment.

Firstly, the dryness of each weather station along the highway was calculated using de Martonne Eq [29] based on temperature and precipitation data. Then, the dryness of the study area was obtained by the inverse distance weight(IDW). Based on the results of the site survey, the vegetation distribution in study area was divided into exuberant area, medium area, and sparse area. The angle between the primary wind direction and the highway was calculated from the wind direction data from the weather station combined with the highway alignment in the design documents. Ultimately, it was divided into three different areas depending on the size of the angle.

#### 3.1.3 Evaluation indicators for vulnerability of the hazard-bearing body

The hazard-bearing body is the object of the hazard-causing factors, so the distribution characteristics of the hazard-bearing body directly determine the degree of hazard. As a special disaster-bearing body, the concave horizontal curve and concave vertical curve of highways have an accumulating effect on wind-sand flow. And the smaller the radius of the curve, the more pronounced is its cumulative effect. This will lead to the higher risk of Windblown sand hazards [30]. Therefore, the radius of concave horizontal curve and radius of concave vertical curve were selected as the evaluation indicators for the vulnerability of the hazard-bearing body. The raster maps of these two indicators were produced by dividing the study area into three different areas depending on the radius.

### 3.2 Weighting

The importance of each indicator to the evaluation results varies, so it is necessary to determine the weight of each indicator. In this paper, the subjective weight was determined by the G1 method, and the objective weight was determined by EWM. The combined weight of each indicator was calculated by the game theory method.

#### 3.2.1 G1 method

The G1 method is a subjective weighting method proposed by Professor Guo Yajun [31]. Compared with the traditional AHP method, it not only makes a lower dependence on experts, but also has a simpler calculation process. The specific calculation steps are as follows.

1. The order of the indicators is determined, and the elements in the set of indicators are compared.
$$x_{1}>x_{2}>,\cdots ,>x_{n}$$ (1)
2. According to the order of the indicators, the relative importance of the indicators is determined by the experts through Eq (2). The reference value is shown in Table 2.
$$r_{i}={\omega \prime }_{i}/{\omega \prime }_{i-1},i=2,3,\cdots ,n$$ (2)
  Where *ω*’_*i*_ and *ω*’_*i*−1_ are the weights of the *i*th and *i*-1th indicators respectively.
3. Calculate the subjective weight of the indicator.
$${\omega \prime }_{n}={\left(1+\sum_{k=2}^{n}\prod_{i=k}^{n}r_{i}\right)}^{-1}$$ (3)
$${\omega \prime }_{i-1}=r_{i}\omega_{i}$$ (4)

**Table 2:** Reference for assignment in *r*_*i*_.

*r*_*i*_	Relative importance
1	Both elements have the same importance compared to each other
1. 2	Compared to the two elements, *x*_*i*_ is slightly more important than *x*_*i*−1_
1. 4	Compared to the two elements, *x*_*i*_ is significantly more important than *x*_*i*−1_
1. 6	Compared to the two elements, *x*_*i*_ is strongly more important than *x*_*i*−1_
1. 8	Compared to the two elements, *x*_*i*_ is extremely more important than *x*_*i*−1_

#### 3.2.2 Entropy weight method (EWM)

EWM is an objective weighting method that uses the variability of the indicator to obtain the weight of the indicator based on the concept of information entropy [32]. The specific calculation steps are as follows.

1. Standardize the indicator:
$$y_{ij}=\left\{\begin{array}{c} \frac{x_{ij}-min\left\{x_{ij}\right\}}{max\left\{x_{ij}\right\}-min\left\{x_{ij}\right\}},\;\text{positive}\;\text{indicator}\\ \frac{min\left\{x_{ij}\right\}-x_{ij}}{max\left\{x_{ij}\right\}-min\left\{x_{ij}\right\}},\;\text{negative}\;\text{indicator} \end{array}\right.$$ (5)
2. Calculate the feature proportion of the first object under the first indicator:
$$p_{ij}=x_{ij}/\sum_{i=1}^{n}y_{ij}$$ (6)
3. Calculate the entropy value of the *j*th indicator:
$$e_{j}=-k\sum_{i=1}^{n}p_{ij}\;\text{ln}\;p_{ij},k=1/\text{ln}\;n$$ (7)
4. Calculate the weight of the *j*th indicator:

#### 3.2.3 Game theory combination weight

Game theory is generally used to obtain equilibrium solutions to two or more conflicts to maximize benefits. Therefore, the game theory can combine subjective and objective weights to make the results more realistic. It has been extensively used in some fields [33–37]. In this paper, the G1 method is combined with the entropy weight method to determine the weight through the SHAP index of game theory. The specific calculation steps are as follows.

1. The subjective weight determined by the G1 method and the objective weight determined by the entropy weight method (EWM).
$$\omega =\alpha_{1}\omega \prime +\alpha_{2}\omega \prime \prime$$ (8)
2. Game theory is used to bring different weight vectors into agreement and compromise. The goal of minimizing the deviation *ω* and *ω*_*k*_ is achieved by optimizing the linear combination coefficient *α*_1_ and *α*_2_.
$$min{\left\|\omega -\omega_{k}\right\|}_{2},\omega_{k}=\omega \prime ,\omega \prime \prime$$ (9)
  According to the differential properties, the first-order derivative condition of the above Eq optimization is as follows:
$$\left\{\begin{array}{c} \alpha_{1}\omega \prime {\left(\omega \prime \right)}^{T}+\alpha_{2}\omega \prime {\left(\omega \prime \prime \right)}^{T}=\omega \prime {\left(\omega \prime \right)}^{T}\\ \alpha_{1}\omega \prime \prime {\left(\omega \prime \right)}^{T}+\alpha_{2}\omega \prime \prime {\left(\omega \prime \prime \right)}^{T}=\omega \prime \prime {\left(\omega \prime \prime \right)}^{T} \end{array}\right.$$ (10)
3. Normalize the linear combination coefficient:
$$\alpha_{k}^{*}=\frac{\alpha_{k}}{\alpha_{1}+\alpha_{2}},k=1,2$$ (11)
4. Calculate the combined weight:
$$\omega =\alpha_{1}^{*}\omega \prime +\alpha_{2}^{*}\omega \prime \prime$$ (12)

### 3.3 Evaluation model

The raster maps of each indicator were standardized and then based on the weight of the indicators, ArcMap10.2 was used to calculate the weighted sum of index layers to obtain the visualization results of danger of the hazard-causing factors, vulnerability of the hazard-forming environment, and vulnerability of the hazard-bearing body. Further grading according to Table 3, and the ultimate the danger map of the hazard-causing factors(*H*), the vulnerability map of the hazard-bearing environment (*E*), and the vulnerability map of the hazard-bearing body (*V*) were obtained, respectively.

**Table 3:** Comment set.

Comment Set	[0,0.2]	[0.2,0.4]	[0.4,0.6]	[0.6,0.8]	[0.8,1]
Level	Very low	Low	Medium	High	Very high

The hazard-causing factors and the hazard-bearing body are the sufficient and necessary conditions for the occurrence of windblown sand hazards respectively, and the hazard-forming environment plays a role in amplifying or shrinking to a certain extent. Even if the vulnerability of the hazard-bearing body is high, the risk of windblown hazards will be very low when the probability of the danger of the hazard-causing factors is zero. Therefore the risk index of windblown sand hazards is calculated through the raster calculator function of ArcGIS by Eq (13). After grading the results according to the natural breaks method, the windblown sand hazards risk map along the highways was obtained.

R=H×E×V (13)

Where R is the risk index of windblown sand hazards. the larger the value of R, the higher the risk of windblown sand hazards.

## 4 Results

### 4.1 Weight determination

Based on the indicator system of the risk assessment of windblown sand hazards along the highway, 13 senior experts from design unit, construction unit, operating unit, and universities were invited to participate in the sort and assignment of the indicators. The subjective and objective weights of the indicators were calculated according to the G1 method and the entropy weight method (EWM) respectively. The combined weight of each indicator was calculated by the game theory method. The optimal linear combination coefficients of hazard-causing factors, hazard-forming environment, and hazard-bearing body evaluation indicators were calculated by Eqs (10) and (11) to be (0.240, 0.760), (0.362, 0.638), (0.433, 0.567). The optimal comprehensive weight is calculated by Eq (12), and the results are shown in Table 4.

**Table 4:** The result of the weight calculation.

	Danger of the disaster-causing factors		Vulnerability of the disaster-bearing environment			Vulnerability of the disaster-bearing body	
	Annual average wind speed	Type of sandy land	Vegetation distribution	Dryness	Angle between the primary wind direction and the highway direction	Radius of concave horizontal curve	Radius of concave vertical curve
G1	0.400	0.600	0.627	0.094	0.279	0.600	0.400
EWM	0.552	0.448	0.413	0.277	0.310	0.526	0.474
Combination weight	0.516	0.484	0.490	0.211	0.299	0.558	0.442

### 4.2 Danger of the hazard-causing factors

**Fig 4:**
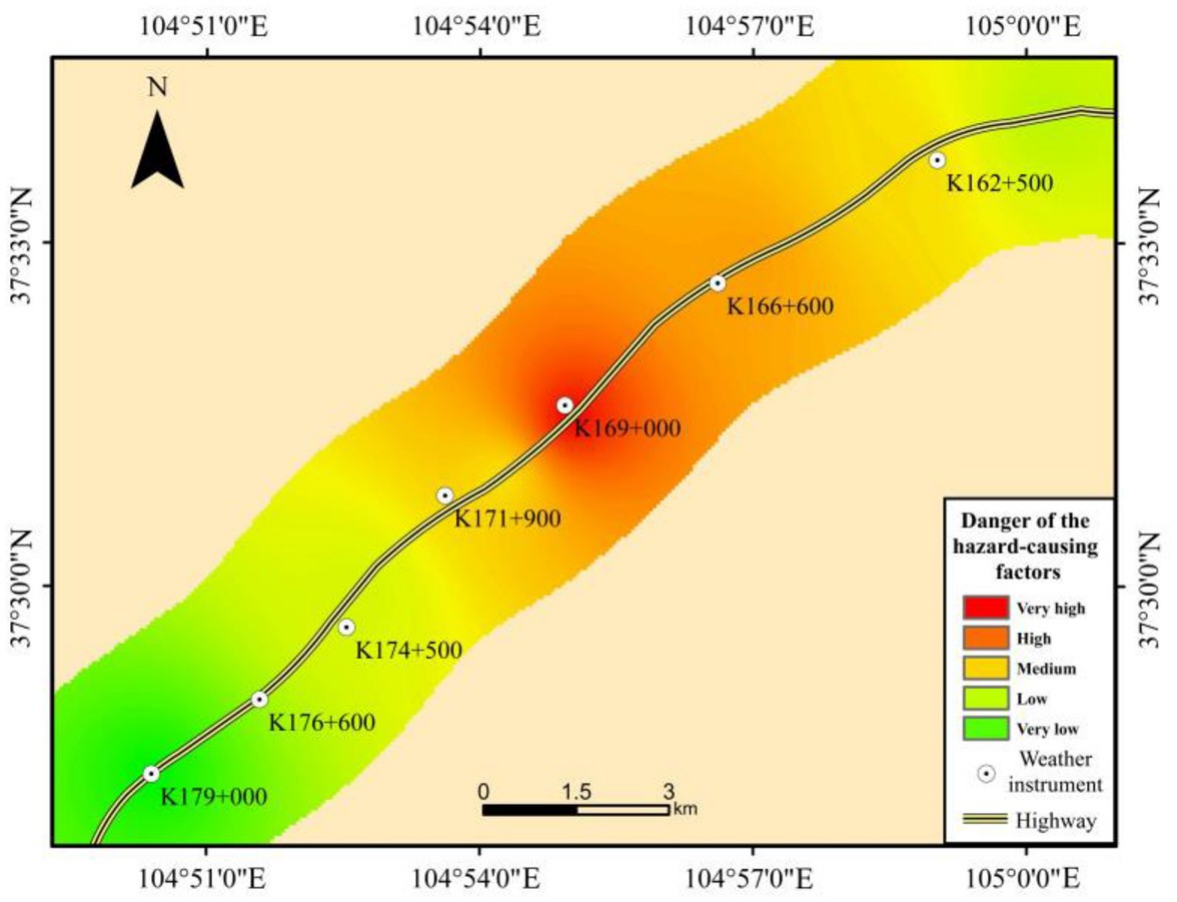
The danger map of the hazard-causing factors.

As shown in Fig 4 and Table 5, the danger of the hazard-causing factors along the Wuhai-Maqin Highway is mainly medium and high, and the length percentage is about 58.9%. This is followed by low and very low danger, with a length percentage of about 31.3%. The smallest length is very high danger, accounting for about 9.8%. From the perspective of spatial distribution, the high and very high danger sections of the hazard-causing factors are mainly distributed in the K164+500 to K170+000 of the Wuhai-Maqin Highway in the hinterland of the Tengger Desert. On the one hand, this is due to the wide distribution of shifting dunes along this section of the highway, which provides ample sand source for windblown sand activity. On the other hand, the annual average wind speed in this area is significantly larger, which provides a strong impetus for windblown sand activity. The low and very low danger sections of the hazard-causing factor are located in areas of flat sandy land and fixed sandy land, where sand sources are sparser and wind speeds are relatively low. The medium danger section of the hazard-causing factor is basically located at the edge of the shifting dunes area. Although windblown sand activity in these areas is weaker than that in the desert hinterland, the windblown sand activity is well developed due to the proximity to the desert hinterland.

**Table 5:** Length percentage on danger grade of the hazard-causing factors.

	Grade	Length percentage(%)
Danger of the hazard-causing factors	Very high	9.8%
	High	22.1%
	Medium	36.8%
	Low	17.6%
	Very low	13.7%

### 4.3 Vulnerability of the hazard-forming environment

**Fig 5:**
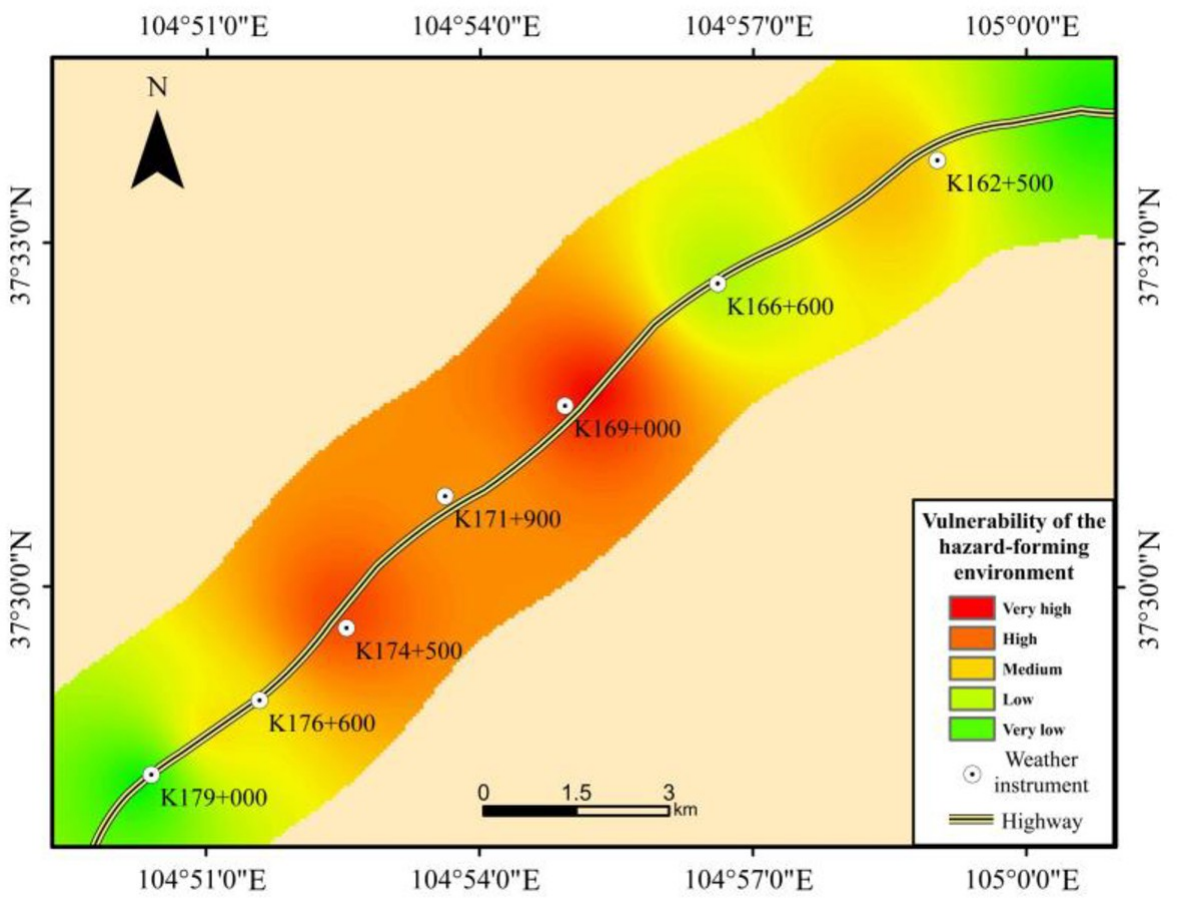
The vulnerability map of the hazard-bearing environment.

As shown in Fig 5 and Table 6, the vulnerability of the hazard-forming environment along the Wuhai-Maqin Highway is mainly medium and high, and the length percentage is about 55.1%; This is followed by low and very low danger, with a length percentage of about 31.4%; The smallest length is very high danger, accounting for about 13.5%. From the perspective of spatial distribution, the high and very high vulnerability sections of the hazard-forming environment are mainly distributed in the K167+600 to K176+600 of the Wuhai-Maqin Highway in Tengger Desert. On the one hand, these sections are in the shifting dunes area, where annual precipitation in these areas is scarce (within 130mm) and evaporation is strong. On the other hand, vegetation cover is extremely low, and the cohesion between the sand grains is almost zero. These provide good conditions for the formation of windblown sand activities. In addition, the intensity of the windblown sand flows acting on the highway is relatively high due to the angle between the primary wind direction and the highway direction is greater than 60°. The low and very low vulnerability sections of the hazard-forming environment are located in areas of flat sandy land and fixed sandy land, where vegetation distribution is exuberant and the relatively high precipitation makes for a moist environment overall. The medium vulnerability sections of the hazard-forming environment is basically located in central and marginal areas of shifting dunes area. Although the vegetation is sparse and the precipitation is also small in the desert hinterland, there will be no strong windblown sand activity due to angle between the primary wind direction and the highway direction are below 40°.

**Table 6:** Length percentage on vulnerability grade of the hazard-bearing environment.

	Grade	Length percentage(%)
Vulnerability of the hazard-forming environment	Very high	13.5%
	High	29.0%
	Medium	26.1%
	Low	15.9%
	Very low	15.5%

### 4.4 Vulnerability of the hazard-bearing body

**Fig 6:**
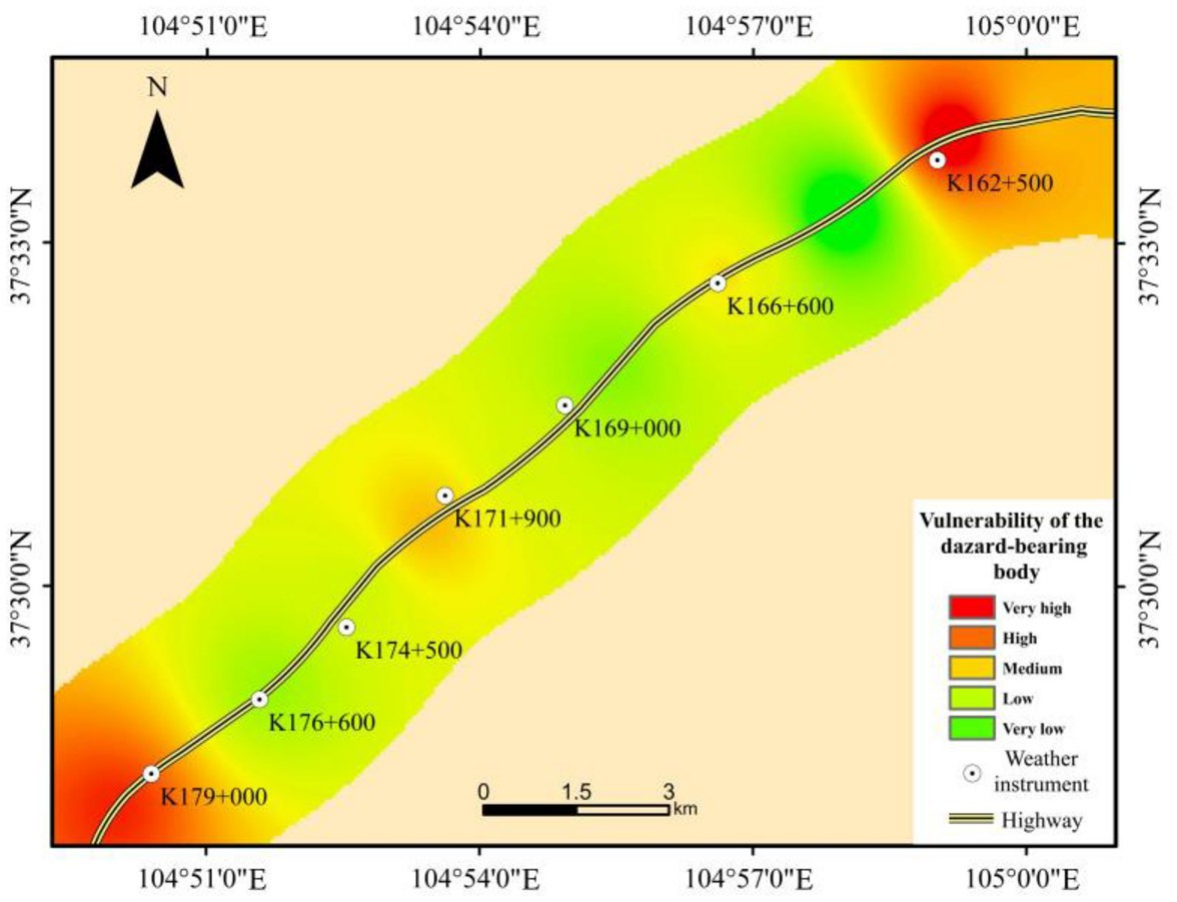
The vulnerability map of thehazard-bearing body.

As shown in Fig 6 and Table 7, the vulnerability of the hazard-bearing body along the Wuhai-Maqin Highway is mainly medium and low, and the length percentage is about 64.2%; This is followed by high and very high danger, with a length percentage of about 28.9%; The smallest length is very low danger, accounting for about 6.9%. From the perspective of spatial distribution, the high and very high vulnerability sections of the hazard-bearing body are mainly distributed in the flat sandy land and fixed sandy land areas. This is mainly due to the use of the small radius of concave horizontal curve in the desert section of the Wuhai-Maqin Highway when connecting with the traffic arteries at both ends in order to avoid photovoltaic power stations and buildings. Most of the highway with medium or lower vulnerability of the hazard-bearing body is located in the shifting dunes area, and these sections adopt large radius of concave horizontal curve and Radius of concave vertical curve.

**Table 7:** Length percentage on vulnerability grade of the hazard-bearing body.

	Grade	Length percentage(%)
Vulnerability of the hazard-bearing body	Very high	14.2%
	High	14.7%
	Medium	37.4%
	Low	26.8%
	Very low	6.9%

### 4.5 Risk of the windblown sand hazards

**Fig 7:**
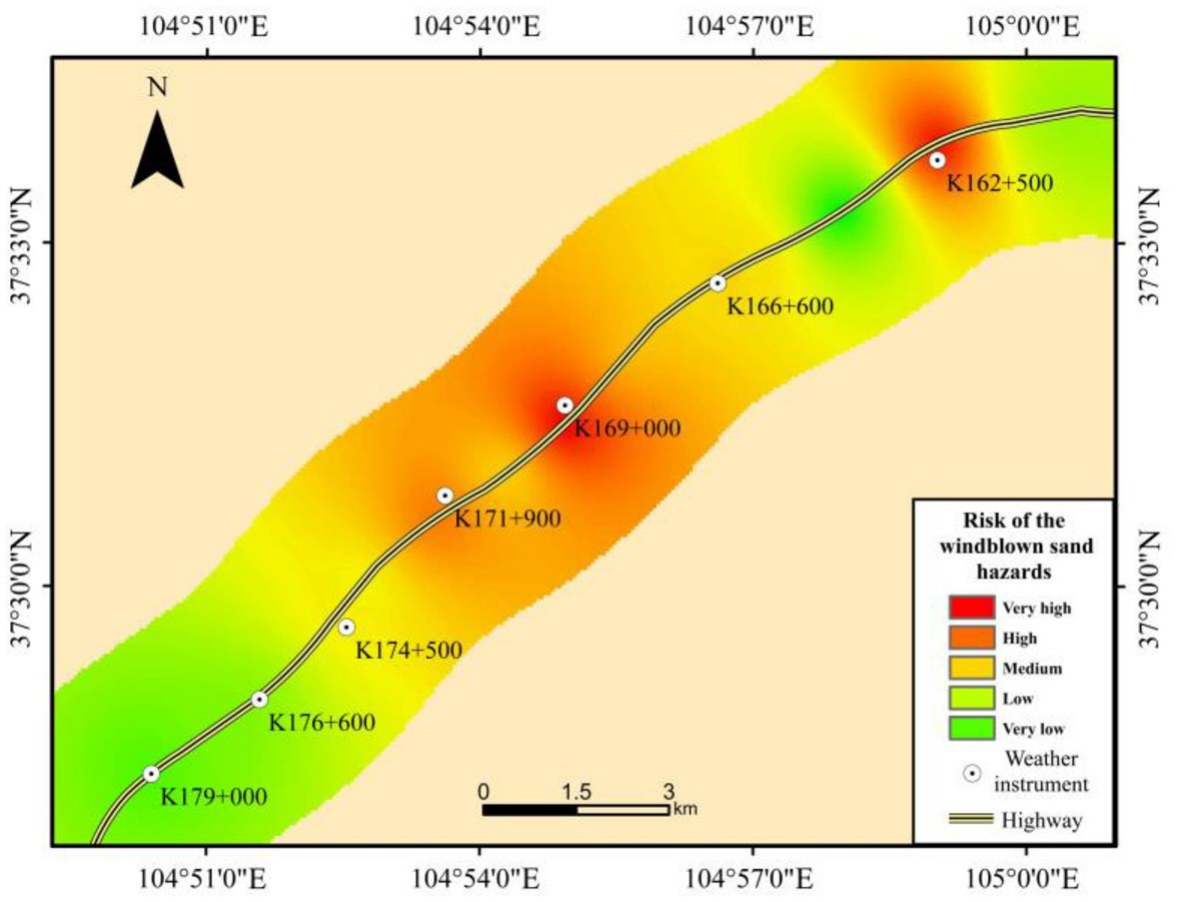
The windblown sand hazards risk map.

As shown in Fig 7 and Table 8, the risk of the windblown sand hazards along the Wuhai-Maqin Highway is mainly medium, low, and very low. High and very high risk windblown sand hazards sections account for only 33.5% of the total length of the highway. From the perspective of spatial distribution, high and very high risk sections of the windblown sand hazards are consist of two main parts. A part of it is located in the hinterland of the shifting dune area, which is not only an abundant sand source and high wind speeds, but also has sparse vegetation and scarce precipitation. Under the influence of the wind, it is easy to form a continued strong wind-sand flow. This is very likely to lead to an accumulation of sand particles on both sides of the highway, or even to sand burial. The other part is located near the small radius of concave horizontal curve of the highway in the flat sandy land area. Due to the proximity to the shifting sand dunes area, the windblown activity is well developed. In addition, the sand is extremely easy to accumulate near the small radius of concave horizontal curve. Therefore, the risk of windblown sand hazards is also relatively high.

**Table 8:** Length percentage on risk grade of the windblown sand hazards.

	Grade	Length percentage(%)
Risk of the windblown sand hazards	Very high	13.2%
	High	20.3%
	Medium	26.9%
	Low	19.2%
	Very low	20.4%

## 5 Validation and discussion

### 5.1 Validation

**Fig 8:**
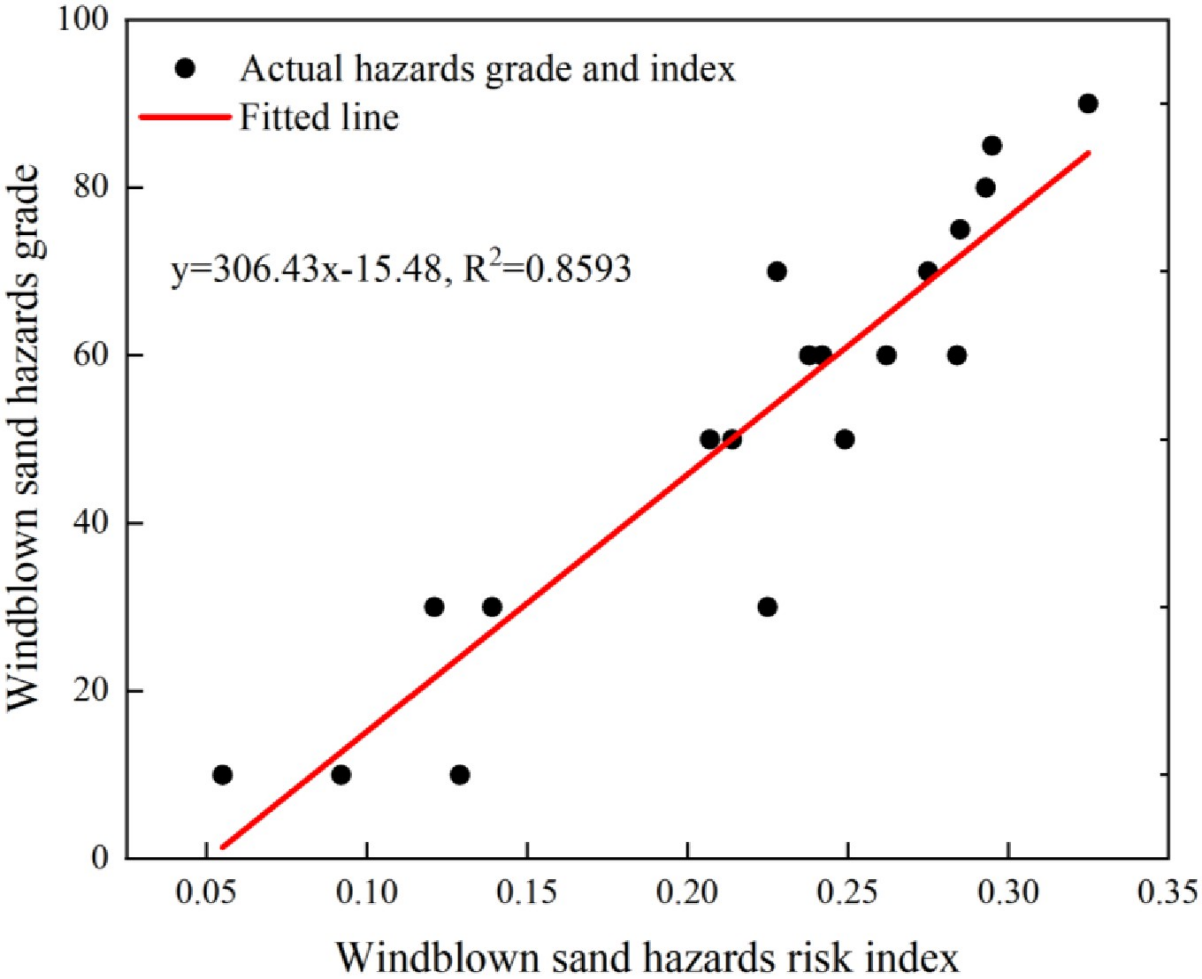
Comparison of the windblown sand hazards risk and Windblown sand hazards grade. Red line is the best linear fit.

The actual windblown sand hazards data along the highways was used to verify the windblown sand hazards risk assessment results. Through site surveys, actual windblown sand hazards data along the Wuhai-Maqin Highway were obtained and classified into five grades from low to high according to (0,20), (20,40), (40,60), (60,80) and (80,100). Then, these were fitted with windblown sand hazards risk index for the corresponding location. As shown in Fig 8, the correlation was up to 85.93%. Therefore, the selection of indicators and the method of GIS-game theory combination weight assessment could reasonably be used to construct the windblown sand hazards risk assessment model along the highways.

### 5.2 Discussion

In previous studies on windblown sand hazards risk assessment along highways and railways, the selection of indicators was not comprehensive enough. Most of the studies [38] take climate and environmental factors as the main evaluation indicators, while ignoring the influence of roads and railways’own attributes on the risk of windblown sand hazards. In the same climate and environment, concave highway alignment tends to accumulate wind-sand flow, which will easier result in higher risk of windblown sand hazards. In this study, considering the influence of highway alignment on windblown sand hazards risk, the radius of concave horizontal curve and the radius of concave vertical curve were used as assessment indicators, which made the indicators system more scientific.

The accuracy of the assessment model is a key issue in the application of risk assessment models. And the accuracy of the assessment results is mainly controlled by the weights of the indicators. In previous studies on assessment, most of the indicator weights are based on the single AHP [39, 40], which is highly influenced by the subjectivity of decision makers. In this study, data information of climate, environment and highways was fully extracted. Combined with the more reasonable game theory combination weight method, the impact of subjective decisions by decision makers is reduced. The accuracy of the model was significantly improved.

Since the distance between weather stations is not very dense, the distribution of wind speed and dryness along the highway drawn by GIS cannot fully accurately reflect the real situation along the highway. In order to further improve the accuracy of the data, more intensive weather stations should be deployed. In addition, as windblown sand hazards along highways vary from region to region, the results of verification using the windblown sand hazard data for one region are one-sided. Therefore, to further verify the credibility of the model, complete windblown sand hazards data from empirical research on other regions are required.

## 6 Conclusions

This paper proposed a windblown sand hazard risk assessment model along the highways based on the combination weighting method of game theory. In the MCDM framework, danger of the hazard-causing factors, vulnerability of the hazard-forming environment, and vulnerability of the hazard-bearing body were the evaluation criteria, and seven indicators were used to establish the index system. With the support of GIS, the danger map of the hazard-causing factors, the vulnerability map of the hazard-forming environment, the vulnerability map of the hazard-bearing body, and the windblown sand hazards risk map were displayed, and the windblown sand hazards risk level was classified into five levels according to the natural breaks method. The game theory combination weighting can make the subjective and objective both achieve the equilibrium and improve the scientificity and reasonability of weight results.

Applying the proposed windblown sand hazards risk assessment model along the highways to Wuhai-Maqin Highway, the evaluation result shows that 33% of the section is above medium risk, and the high and very high risk highway sections are mainly distributed in the hinterland of the shifting dune area and near the horizontal curve with a small radius in the flat sandy land area. Validation results show the model performed well.

The research results can provide valuable information for windblown sand hazards risk management, windblown sand hazards prevention, and risk reduction along the highways. However, there are two main limitations in this study. First, the distance between weather stations is not very dense. More intensive weather stations should be deployed in further research. Second, the proposed model needs to be validated in areas where windblown sand hazards occur frequently along other highways.
